# Lexicon and attention-based named entity recognition for kiwifruit diseases and pests: A Deep learning approach

**DOI:** 10.3389/fpls.2022.1053449

**Published:** 2022-11-17

**Authors:** Lilin Zhang, Xiaolin Nie, Mingmei Zhang, Mingyang Gu, Violette Geissen, Coen J. Ritsema, Dangdang Niu, Hongming Zhang

**Affiliations:** ^1^ College of Information Engineering, Northwest Agricultural and Forestry (A&F) University, Yangling, China; ^2^ Soil Physics and Land Management Group, Wageningen University, Wageningen, Netherlands

**Keywords:** intelligent farming for diseases recognition, Chinese named entity recognition, kiwifruit diseases and pests, data mining, lexicon, Criss-cross attention, deep learning, machine learning

## Abstract

Named Entity Recognition (NER) is a crucial step in mining information from massive agricultural texts, which is required in the construction of many knowledge-based agricultural support systems, such as agricultural technology question answering systems. The vital domain characteristics of Chinese agricultural text cause the Chinese NER (CNER) in kiwifruit diseases and pests to suffer from the insensitivity of common word segmentation tools to kiwifruit-related texts and the feature extraction capability of the sequence encoding layer being challenged. In order to alleviate the above problems, effectively mine information from kiwifruit-related texts to provide support for agricultural support systems such as agricultural question answering systems, this study constructed a novel Chinese agricultural NER (CANER) model KIWINER by statistics-based new word detection and two novel modules, AttSoftlexicon (Criss-cross attention-based Softlexicon) and PCAT (Parallel connection criss-cross attention), proposed in this paper. Specifically, new words were detected to improve the adaptability of word segmentation tools to kiwifruit-related texts, thereby constructing a kiwifruit lexicon. The AttSoftlexicon integrates word information into the model and makes full use of the word information with the help of Criss-cross attention network (CCNet). And the PCAT improves the feature extraction ability of sequence encoding layer through CCNet and parallel connection structure. The performance of KIWINER was evaluated on four datasets, namely KIWID (Self-annotated), Boson, ClueNER, and People’s Daily, which achieved optimal F_1_-scores of 88.94%, 85.13%, 80.52%, and 92.82%, respectively. Experimental results in many aspects illustrated that methods proposed in this paper can effectively improve the recognition effect of kiwifruit diseases and pests named entities, especially for diseases and pests with strong domain characteristics

## 1 Introduction

Kiwifruit is one of the economic sources of the planting industry in China, but owing to the impact of pests and diseases, the overall level of kiwifruit quality in China is not high at present (Jiang and Zong, 2020). Chinese named entity recognition in the field of agriculture aims to recognize the boundaries and categories of agriculture-related entities from unstructured agricultural texts, such as diseases, pests, and pesticides (Guo et al., 2020). This is a key technology in the automatic mining of knowledge from very large Chinese agricultural texts and is the basis for downstream tasks such as building agricultural knowledge graphs and constructing agricultural intelligent question-and-answer (Q&A) systems (Drury and Roche, 2019; Guo et al., 2020). Therefore, accurate recognition of named entities in the field of kiwifruit plays an important role in ensuring the healthy development of the industry, plant protection, and convenience for agricultural workers.

Traditional NER methods can be divided into rule-based, dictionary-matching-based, and machine-learning-based approaches (Guo et al., 2020). Although each approach can achieve good results, they rely heavily on time- and energy-consuming pattern matching and feature engineering and have poor generalization ability. Through the application of deep learning in the field of NER and other fields (Chiu and Nichols, 2016; Bhatti et al., 2020b), researchers have developed various techniques for medical science (Zhao et al., 2019; Bhatti et al., 2021; Nawaz et al., 2021), cyber security (Li T et al., 2020), agriculture (Biswas and Sharan, 2021), social media (Aguilar et al., 2017) and environmental science (Bhatti et al., 2020a; Aamir et al., 2021; Galvan et al., 2022). In the field of Chinese NER (CNER), because sentences in Chinese texts are not naturally separated, unlike sentences in English, there is no obvious border symbol. Therefore, the first step in many original deep-learning-based CNER methods is to segment the text using word segmentation tools (Yang et al., 2016; He and Sun, 2017). With the development of research on CNER, many researches show that the character-based CNER model avoids segmentation errors and makes it more suitable than the word based model. (Jingzhou and Houfeng, 2008; Liu et al., 2010). However, in order to avoid the problem of segmentation errors, the character based CNER model cannot use Chinese word information. Recently many researchers have realized that word information will play a positive role in the correct recognition of Chinese entity boundaries. Therefore, lexicon-based CNER models have been widely used in recent years. For example, Zhang and Yang (2018) introduced the lattice long short-term memory model (Lattice-LSTM) based on a lexicon, allowing character-level and word-level information corresponding to the characters to be encoded simultaneously. Peng et al. (2020) proposed the Softlexicon method to integrate word information into the NER model by simply adjusting the character representation layer. The lexicon based model, with the help of the public lexicon, achieves better results than the purely character based model (Peng et al., 2020). For example, when the lexicon based model recognizes the Chinese entity “长江大桥” (Yangtze River Bridge), words such as “长江” (Yangtze River), “大桥” (Bridge), and “长江大桥” (Yangtze River Bridge) in the lexicon can help eliminate the ambiguity of potentially related named entities in the context, such as the person name “江大桥” (Daqiao Jiang) (Zhang and Yang, 2018).

For CNER in the field of agriculture (CANER). The lexicon-based method makes good use of character information and word information, so using them to solve the CANER problem may be a theoretically feasible solution too. However, there is currently no open-source lexicon in the field of agriculture, and manual lexicon construction is labor-intensive. If the lexicon is built through automatic word segmentation, the existing word segmentation tools face the problem of word segmentation errors caused by insensitive word segmentation. For example, farm chemicals entities such as “速乐硼、辛硫磷乳油” (solubor, phoxim) and kiwifruit variety entities “中华猕猴桃、红心猕猴桃” (Actinidia chinensis Planch., red-fleshed kiwi), which exist in kiwifruit-related texts, have strong domain characteristics, and these will make the word segmentation tool insensitive in the form of out-of-vocabulary (OOV) words. Therefore, many CANER methods are still character-based models (Guo et al., 2020; Zhao et al., 2021; Guo et al., 2022), and the use of word information is hindered by word segmentation errors. As for the sequence coding layer of recently CANER model, bidirectional long short-term memory (BiLSTM) is still the mainstream deep learning method, which can memorize long-text sequence features in theory (Liu et al., 2020; Zhao et al., 2021). However, the contextual feature extraction ability of BiLSTM has the following limitations. First, with an increase in sentence length, the feature extraction ability of BiLSTM will decline (Li Y et al., 2020). Second, BiLSTM makes each character contribute equally to the task (Guo et al., 2020), but the contribution of different types of characters in agricultural texts to the task is certainly different. Third, the strong domain features of kiwifruit-related text, particularly farm chemical-related entities, disease-related entities, and pest-related entities, pose a challenge to the feature extraction ability of BiLSTM. In summary, deep learning-based methods for CANER in the field of kiwifruit diseases and pests face the following problems: The use of word information is hampered by OOV problem in the process of lexicon construction. And the contextual information capture capability of the sequence encoding layer needs to be further improved.

This research proposes a lexicon-based CANER model KIWINER on the basis of bidirectional long short term memory and conditional random field model (BiLSTM-CRF). The objectives of KIWINER are to take measures to solve the above problems in the end of the previous paragraph, that is, to integrate the word information containing domain features into the model, improve the model feature extraction ability, and ultimately provide support for the construction of the kiwifruit Q&A system. Specifically, KIWINER improves the recognition quality through statistics-based new word detection, AttSoftlexicon, and PCAT. First, statistics-based new word detection is innovatively used to detect new words in kiwifruit-related text corpora, thereby improving the adaptability of word segmentation tools to kiwifruit-related texts and reducing the impact of word segmentation errors on the lexicon construction process; Second, through the AttSoftlexicon method proposed in this paper, based on Softlexicon (Peng et al., 2020) and CCNet (Huang et al., 2019), the character and word information in the lexicon are integrated into the model, and the position information of the character in the corresponding words can be fully utilized with the help of CCNet (Huang et al., 2019); Third, a novel module parallel connection criss-cross attention network (PCAT) is proposed to improve the contextual feature extraction ability of BiLSTM. PCAT assigns different weights to different characters according to their correlation and constructs a parallel structure through convolutional layers with different filter sizes to obtain richer semantic information. Additionally, this study collected publicly available textual information and constructed a kiwifruit NER dataset consisting of 17809 entities across six categories. Previous CANER methods based on machine learning, such as CRF (Li et al., 2017), rely on manual features or rules, which are time-consuming and unable to process a large number of complex agricultural texts (Guo et al., 2020). The CANER methods such as Att-BiLSTM-CRF (Zhao et al., 2021) use the deep learning method to reduce the work of designing feature extractors for each problem and solve the above problems. Compared with the popular CANER methods based on deep learning, our proposed KIWINER alleviates the OOV problem through new word detection, and makes full use of lexical information and agricultural features in addition to character information through AttSoftlexicon and PCAT, so the feature extraction ability of deep learning model is effectively improved. We also use KIWINER and five typical CNER models and two popular CANER models for comparative experiments, and the KIWINER model yields better performance.

The remainder of this paper is organized as follows. The materials used in this study and the methods proposed in this paper is discussed in detail in section 2. Section 2 also introduces the experimental parameters, dataset division, evaluation metrics, and the experimental environment. The experimental details and results are presented in Section 3. The discussion of this study is presented in section 4. Finally, the conclusions are presented in Section 5.

## 2 Materials and methods

**Figure 1:**
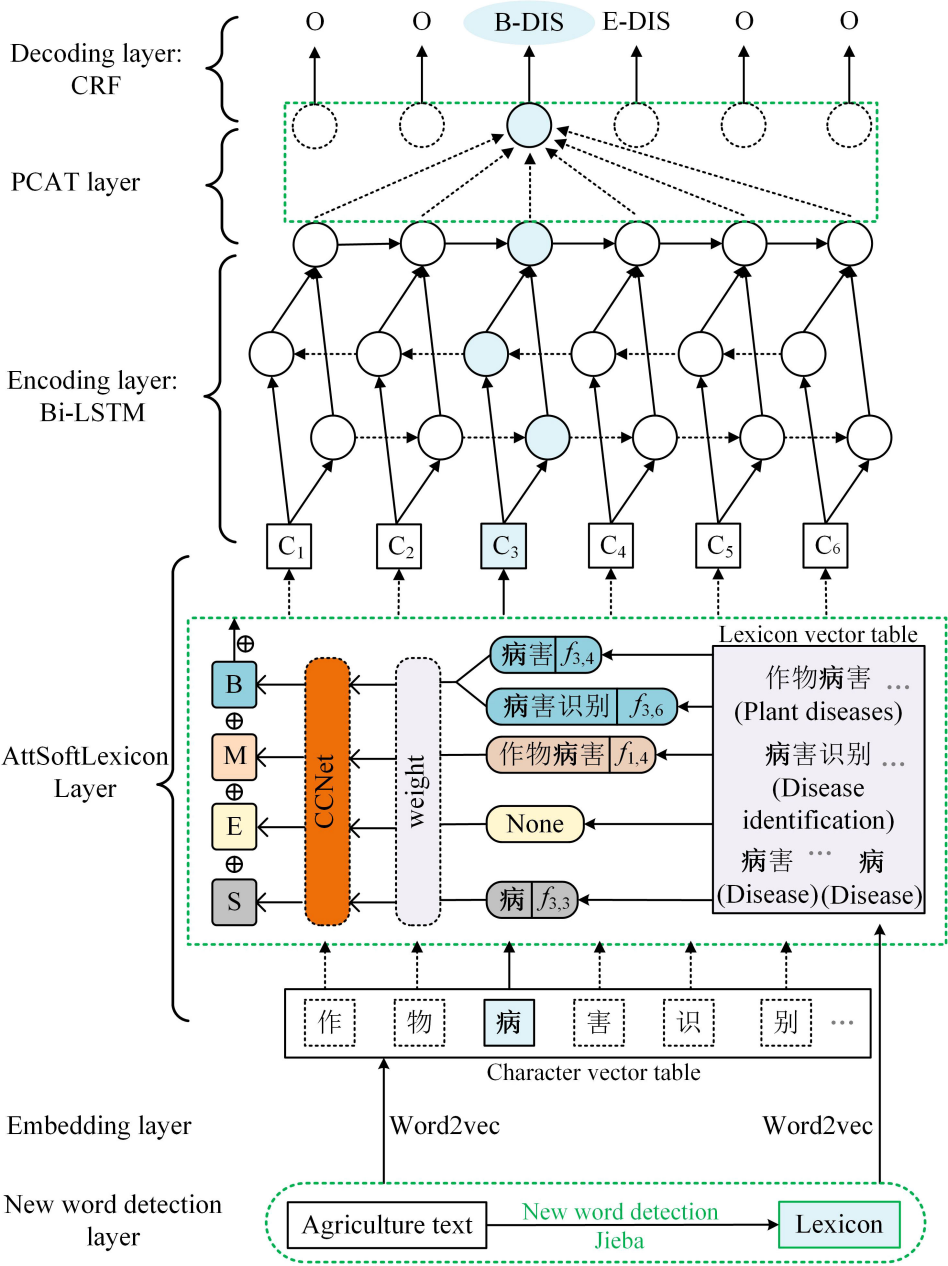
The architecture of KIWINER.

The overall architecture of KIWINER, shown in **Figure 1**, indicates that the model contains six layers and uses BiLSTM-CRF as the basic framework. This section first introduces the experimental materials. Then this section focuses on the implementation details of the new word detection layer, embedding layer, CCNet, and AttSoftlexicon and PCAT proposed in this paper. Details of the BiLSTM and CRF layers can be found in (Huang et al., 2015).

### 2.1 Materials

To solve the problem of the limited public NER dataset for CANER, a new kiwifruit-related annotated corpus, named KIWID, was collected and annotated under the guidance of plant protection experts from Northwest A&F University.

#### 2.1.1 Corpus collection

To ensure the quality of data, this study collected public information on kiwifruit diseases and pests from the official websites of trusted research institutions and Baidu Encyclopedia. Preprocessing was applied to remove non-useful content, such as webpage tags, links, and special characters contained in the corpus. Finally, a corpus (Corpus A of kiwifruit) containing 61103 sentences for training character vectors and detecting new words was obtained.

#### 2.1.2 Corpus tagging

We selected 12477 sentences from Corpus A to form Corpus B. Under the guidance of plant experts from Northwest A&F University, six types of kiwifruit-related entities were labeled, as shown in **Table 1**. Therefore, this study used the BMES (Ratinov and Roth, 2009) tagging scheme to tag Corpus B, where B, M, E, and S represent the beginning, middle, and end of an entity, and a single-word entity, respectively. To ensure annotation quality, the manual annotation method was adopted. Finally, the kiwifruit-related dataset KIWID containing 17809 entities was obtained, and the statistical information of KIWID is presented in the last column of **Table 1**.

**Table 1:** Statistics of KIWID.

Category (Symbol)	Category definition	Examples	Numbers
Varieties (KIWI)	Names of different varieties of kiwifruit.	陇南猕猴桃 (Longnan kiwi)	3763
Disease (DIS)	Diseases of kiwifruit.	叶枯病 (Leaf blight)	561
Pest (PEST)	Pests of kiwifruit.	叶蝉 (Leaf cicada)	1247
Part (PART)	Diseases harming kiwifruit parts.	叶片 (Leaves)、枝干(branches)	5521
Farm chemical (MED)	Farm chemicals.	多菌灵 (Carbendazim)	907
Place (LOC)	Distribution area of kiwifruit	陕西 (Shaanxi)	5090

#### 2.1.3 Analysis of Corpus features

(1) Contains several specialized vocabulary terms.

Entities involved in agricultural diseases and pests such as farm chemicals entities, pest entities, plant disease entities, and varieties entities are annotated in the corpus, such as “二甲吗啉” (dimethomorph), “联苯菊酯” (bifenthrin), “介壳虫” (scale insect), and “斑点病” (scab). Such words usually do not appear in the built-in dictionaries of common word-segmentation tools and have strong domain characteristics. Therefore, most word segmentation tools have poor adaptability to these specialized terms, leading to a greater likelihood of word segmentation errors. If the word information in the lexicon constructed by automatic word segmentation is introduced into the CANER model, the accuracy of the model may be significantly affected by word segmentation errors.

(2) Number of entities is unevenly distributed.

As shown in **Table 1**, there are differences in the number of different types of entities. The same problem exists not only in agriculture (Guo et al., 2022) but also in clinical medicine (Kong et al., 2021). The uneven distribution of the number of entities introduces challenges to the feature extraction ability of the CANER models.

(3) Entities nested within each other

Nested named entities are a common problem in the field of NER in the task of identifying kiwifruit-related entities. For example, there are two entities nested in “中华猕猴桃” (Actinidia chinensis Planch.), which are the location entity “中华” (China) and the plant entity “猕猴桃” (kiwifruit). First, this leads to errors in word segmentation. For example, Jieba’s word segmentation result of “中华猕猴桃” (Actinidia chinensis Planch.) is “中华 猕猴桃” (China kiwifruit). If the lexicon for the NER model contains incorrect word segmentation information, it provides misleading information for the identification of entity boundaries. Moreover, the phenomenon of nested entities also increases the difficulty of entity recognition and introduces challenges to the feature extraction ability of the model.

### 2.2 New word detection layer

New word detection can identify OOV words and add them to the built-in dictionary of the word segmentation tool, thus improving the effect of common word segmentation tools (Du et al., 2016). Currently, new word detection is either rule-based (Huiming et al., 2003), statistics-based (Jin and Tanaka-Ishii, 2006), or based on both rules and statistics (Zheng and Wen-Hua, 2002). Methods that rely entirely or partly on rules rely on a manually built rule base. Although the rule base is helpful in improving the effectiveness of new word detection, the construction process is complex and time-consuming, and domain transferability is poor. As a result, this study adopts a statistics-based new word detection method. Corpus A was first segmented into strings using the *N-gram* method, and the garbage strings were then filtered in turn according to the three statistics of word frequency (*WF*), mutual information (*MI*), and contextual entropy (*CE*) of the strings. Subsequently, a new word set was obtained. This new word set was then added to the built-in dictionary of Jieba to improve its applicability to kiwifruit-related texts. Finally, the kiwifruit lexicon was constructed through the word segmentation of Corpus B by Jieba. This section first introduces the methods related to new word detection, and then introduces the lexicon construction process.

#### 2.2.1 *N-gram* Word segmentation

**Figure 2:**
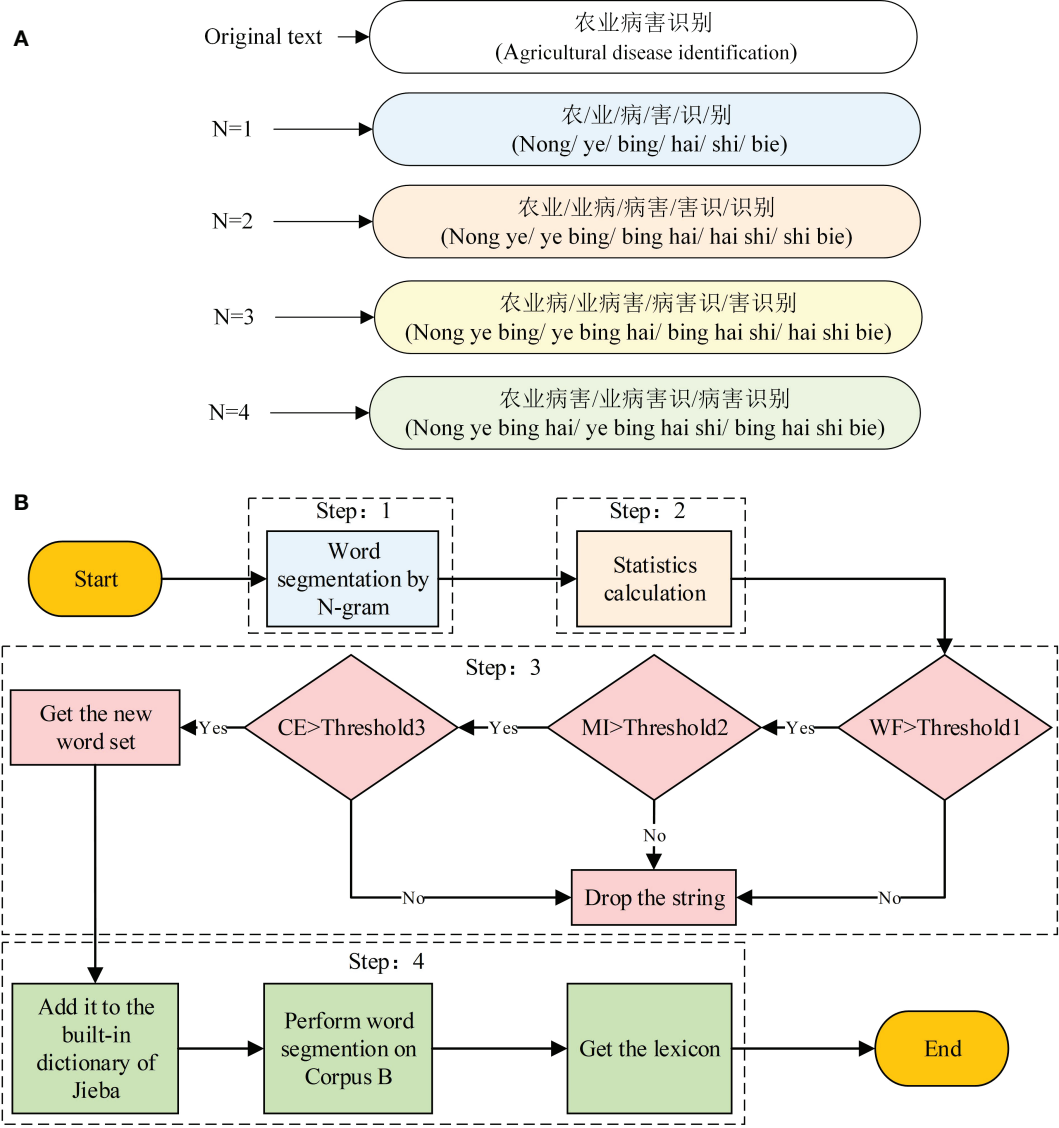
**(A, B)** Lexicon construction process.

The basic idea of *N-gram* word segmentation is to use a fixed window of length *n* to segment the sentence. After segmentation, each string of size *N* is called a “gram.” For example, the 2-gram segmentation result of the sentence “农业病害识别” (agricultural disease identification) is “农业/业病/病害/害识/识别” (nong ye/ye bing/bing hai/hai shi/shi bie). Other examples are shown in **Figure 2A**.

#### 2.2.2 Mutual information

The concept of *MI* originates from information theory and is commonly used to measure how consistently two patterns occur together in a corpus (Ye et al., 2013). The *MI* value is derived from the log-likelihood ratio of the joint probability of patterns *A* and *B* over the individual probabilities of patterns *A* and *B*, as shown in Equation (1). If Chinese strings *w_1_* and *w_2_* in the same dataset appear as a whole string *w_12_*, the probability is *p(w_12_)*, and the probabilities of the two strings appearing alone are *p(w_1_)* and *p(w_2_)*, respectively. The *MI* value was calculated using formula (2). The higher the *MI* value of the two strings, the more likely they are to be combined into meaningful words.


(1) $$MI(x,y)=\log_{2}\frac{p(x,y)}{p(x)p(y)},$$

(2) $$MI({\text{w}}_{1},w_{2})=\log_{2}\frac{p(w_{12})}{p(w_{1})p(w_{2})}.$$

#### 2.2.3 Contextual entropy

*CE* is an external statistic proposed by (Huang and Powers, 2003), that can be used to measure the probability of whether a string is a meaningful word. It measures the randomness of the left and right adjacent characters of a string, that is, the left and right contextual entropies. Compared with a Chinese string with no practical meaning, a Chinese word with a practical meaning has a wider application scenario. Thus, the randomness of the set of left and right adjacent characters will be higher. Therefore, a higher *CE* value for a Chinese string indicates a greater probability that the string has a practical meaning. In the Chinese new word detection task, the *CE* accurately reflects the probability that a string is a meaningful word. The *CE* value was calculated using Equations (3) and (4):


(3) $${\text{E}}_{l}(w)=-\sum_{{\text{w}}_{l}\in S_{l}}P(w_{l}|w)\times \log_{2}P(w_{l}|w),$$

(4) $${\text{E}}_{r}(w)=-\sum_{w_{r}\in S_{r}}P(w_{r}|w)\times \log_{2}P(w_{r}|w),$$

where *p(w_l_|w)* represents the probability that the left-adjacent character of *w* is character *w_l_*, *p(w_r_|w)* represents the probability that the right-adjacent character of *w* is character *w_r_*, *S_l_* represents all left-adjacent characters of *w*, and *S_r_* represents all right-adjacent characters of *w*.

#### 2.2.4 Lexicon construction

The lexicon construction process occurs in four steps, as illustrated in **Figure 2B**).

Step 1: Apply the *N-gram* word segmentation method to segment corpus A and obtain candidate strings with *N* = 2, 3, and 4.

Step 2: Calculate the statistics for each string. Compute the *WF*, *MI*, and *CE* values for each candidate string.

Step 3: Set the corresponding thresholds for *WF*, *MI*, and *CE*, named *Threshold1*, *Threshold2*, and *Threshold3*, respectively, and filter the candidate strings to obtain a new set of words. To avoid the omission of low-frequency new words, we set the *WF* threshold to 5, *MI* threshold to 3.9, and *CE* threshold to 2.7.

Step 4: Add the new word set obtained in Step 3 to the built-in dictionary of Jieba and perform word segmentation on Corpus B to obtain the kiwifruit lexicon for NER.

### 2.3 Embedding Llayer

For a character-based CNER model, discrete text sequences are converted into low-dimensional densely distributed embedded representations, allowing the model to learn more semantic knowledge and improve its performance (Guo et al., 2020). As shown in **Figure 1**, to obtain a high-quality embedded representation and make good use of the information in the corpus, Word2vec-CBOW (Mikolov et al., 2013) was used to train Corpus A in character form and transform the resulting agricultural lexicon into vectors. The input sequence of length *n* is *s=(c_1_, c_2_, c_3_,……,c_n_)∈V_c_*, where *V_c_* is the word set (including characters), and each word is represented by a trained dense vector $x_{i}^{c}=e^{c}(c_{i})$ , where *e^c^* denotes the word embedding lookup table.

### 2.4 CCNet

**Figure 3:**
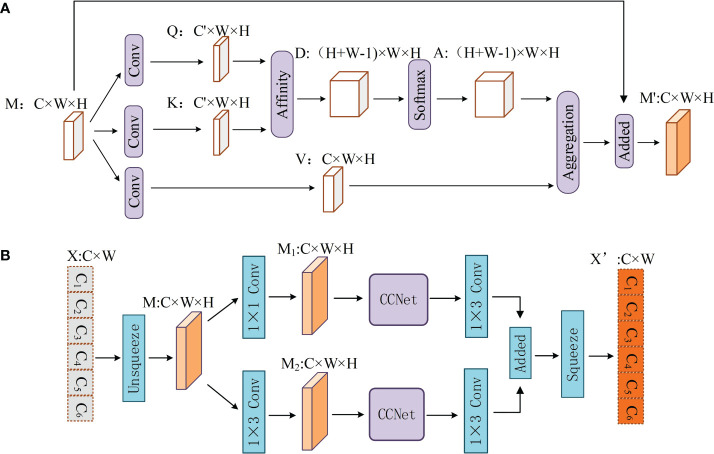
**(A, B)** Structure of CCNet and PCAT.

CCNet (Huang et al., 2019) is often used in semantic segmentation to aggregate contextual information from all pixels to obtain dense contextual information. This study considered the use of CCNet for text feature extraction. The overall structure of the CCNet is shown in **Figure 3A**.

Given a feature map *M∈R^C×W×H^*, CCNet first generates two feature maps *Q* and *K* by applying two convolutional layers with a filter size of 1×1 on the feature map *M*. *{Q, K}∈R^C’×W×H^*, where *C’* is the number of channels of *Q* and *K*, which is less than *C* for dimension reduction. Another convolutional layer with filters of size 1×1 is applied on *M* to generate *V∈R^C×W×H^*. *Q_u_∈R^C’^* is the vector for each position *u* in the spatial dimension of the feature map *Q*. And vector set *Ω_u_∈R^(H+W-1)^×C’* is obtained by extracting feature vectors from *K* which are in the same row with position *u*. Then, CCNet can obtain *D∈R^(H+W-1)×W×H^*, which represents the degree of correlation between features *Q_u_* and *Ω_i,u_* (i=[1,…,|Ω_u_|]) by the affinity operation, which is defined as follows:


(5) $$d_{i,u}=Q_{u}\Omega_{i,u}^{T}$$

where *d_i,u_∈D*. Feature map *A* is then obtained by applying a softmax layer on *D* over the channel dimension. CCNet can also obtain vector *V_u_∈R^C^* and set *θ_u_∈R^(H+W-1)×C^*. The set *θ_u_* is a collection of feature vectors in *V* that are in the same row as position *u*. Finally, the contextual information is collected by the aggregation operation:


(6) Mu'=∑i∈|θu|Ai,uθi,u+Mu

where *M_u’_* is a feature vector in the output feature maps *M’∈R^C×W×H^* at position *u*, and *A_i,u_* is a scalar value at channel *i* and position *u* in *A*. Contextual information is added to local feature *M* to enhance the local features and augment the pixel-wise representation.

### 2.5 Criss-cross attention based Softlexicon layer

One of the tasks of CANER is to recognize the boundaries of agricultural entities, and word segmentation information provides good guidance for identifying entity boundaries. However, CANER is affected by the strong domain characteristics of agricultural texts and the uneven distribution of entity categories (Guo et al., 2020). Adding more pre-training information will help the model learn more agricultural characteristics, thus reducing the impact of the aforementioned problems. Therefore, this paper proposes an AttSoftlexicon based on Softlexicon (Peng et al., 2020) and CCNet (Huang et al., 2019), and integrates the word information in the lexicon into character representation, which helps the model to learn more kiwifruit text features.

Assume that the input sequence is *s={c_1_, c_2_,…, c_n_}*, and *w_i,j_* denotes its subsequence *{c_i_, c_i+1_,…, c_j_}*. The first step is lexicon matching. Each character is matched from a lexicon to all words containing the character. According to the position of each character *c_i_* in the different matched words (beginning, middle, end, or one-character word), the words matched by a character were divided into four-word sets *B(c_i_)*, *M(c_i_)*, *E(c_i_)*, and *S(c_i_)*. The set construction method is shown in formula (7)-(10).


(7) $$B(c_{i})=\{w_{i,k},\forall w_{i,k}\in L,i<k\leq n\},$$

(8) $$M(c_{i})=\{w_{j,k},\forall w_{j,k}\in L,1\leq j<i<k\leq n\},$$

(9) $$E(c_{i})=\{w_{j,i},\forall w_{j,i}\in L,1\leq j<i\},$$

(10) $$S(c_{i})=\{c_{i},\exists c_{i}\in L\}.$$

**Figure 4:**
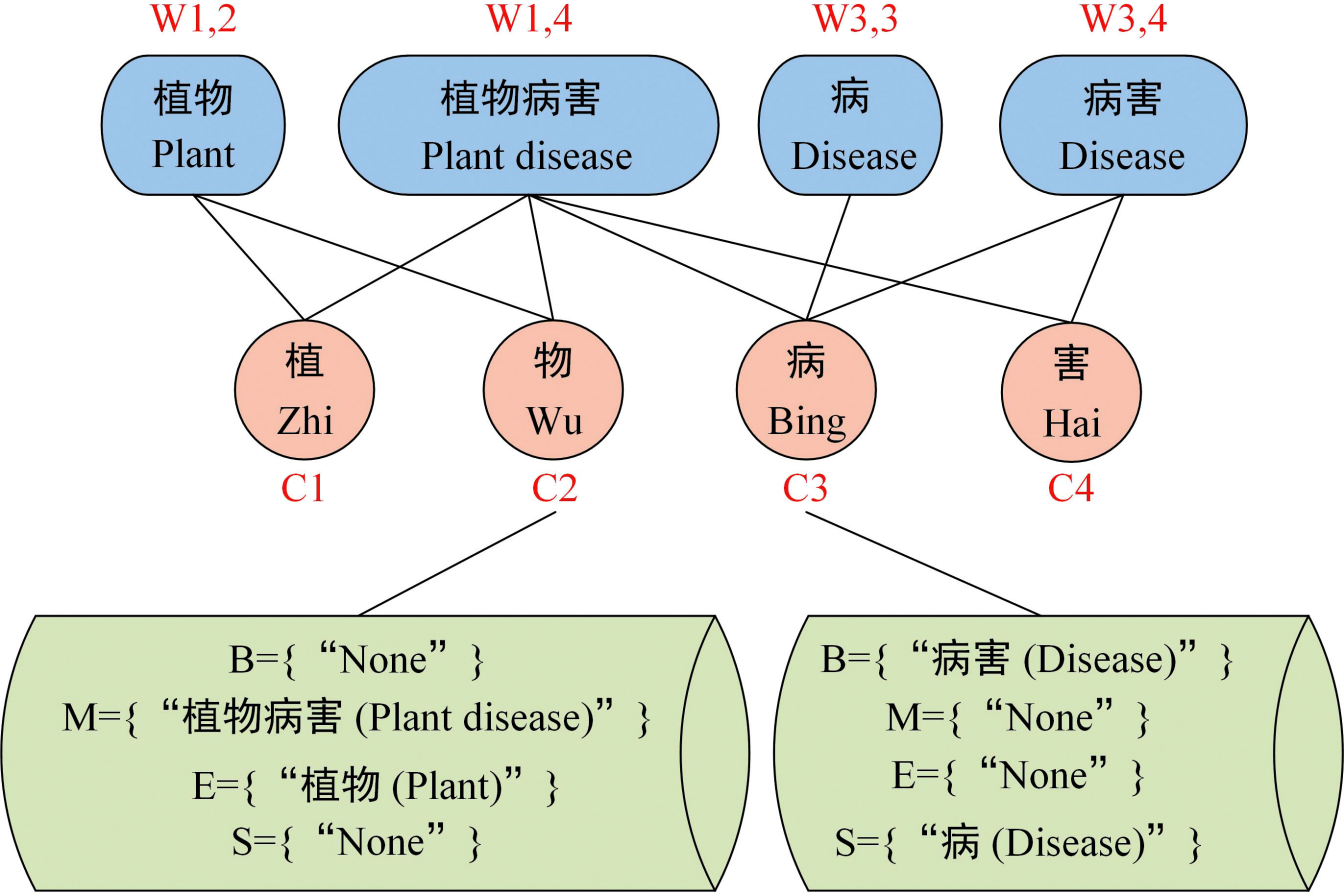
Example of lexicon matching.

As shown in formula (7)-(10), *L* denotes the lexicon, and *w* represents the words matched in the lexicon. If a word set of characters is empty, it is represented as *{None}*. Taking the input sequence “植物病害” (plant disease) as an example, the character “物” (matter) is matched with the pre-constructed lexicon, and the two words “植物病害” (plant disease) and “植物” (plant) are matched, and the four word sets corresponding to the character “物” (matter) are formed: *B={“None”}*, *M={“植物病害”}*, E={“植物”}, *S={“None”}*. The character “病” (disease) is matched with the pre-constructed lexicon, and the two words “病害” (disease and pest) and “病” (disease) are matched, and the four-word sets corresponding to the character “病” (disease) are formed: *B={“病害”}*, *M={“None”}*, *E={“None”}*, *S={“病”}*, as shown in **Figure 4**. To integrate the word set information matched to each character into the corresponding character representation, the statistics-based static weighting method in Softlexicon (Peng et al., 2020) was used, where the frequency reflects the importance of the word.

The weighting method is given by formulae (11) and (12), where *z(w)* is the frequency with which a lexicon word w occurs in the statistical data and *e^w^* is the word embedding lookup table. The weighted representation of word set *S* is obtained as follows:


(11) $$v^{s}(S)=\frac{4}{Z}\sum_{w\in S}z(w)e^{w}(w),$$

Where:


(12) $$Z=\sum_{w\in B\cup M\cup E\cup S}z(w).$$

In the last step, the original Softlexicon (Peng et al., 2020) combines the representations of four-word sets into the fix-dimensional feature and adds it to the representation of each character, as shown in formulae (13) and (14).


(13) $$V=[v^{s}(B);v^{s}(M);v^{s}(E);v^{s}(S)],$$

(14) $$x^{c}\leftarrow [x^{c};V].$$

The original Softlexicon (Peng et al., 2020) designed four-word sets to take advantage of these four types of positional information. However, it only weighs the words in each word set according to the word frequency and does not distinguish the importance of different word sets. This does not allow the model to distinguish the four positions of the characters in the matched words.

CCNet (Huang et al., 2019) showed a strong contextual relationship extraction ability in the semantic segmentation task. Therefore, to make full use of these four types of position information, this study uses CCNet to learn the weights for different word sets, as shown in the formula (15). First, CCNet processes the representation of these four sets and automatically assigns weights to them based on the relationship between them. It is then transformed into a vector of 1×4 through *q*. Finally, the weight vector *a_i_* (i∈[1,4]) with a value range of (0, 1) is obtained through the sigmoid function. *a_i_* is a weight matrix of dimensions 1×4, where the four values represent the importance of the four word sets. As shown in formula (16), the four-word set representations are weighted and merged into the character representation.


(15) $$a_{i}=sigmoid(qCCNet(V))$$

(16) $$x^{c}=[x^{c};a_{1}v^{s}(B);a_{2}v^{s}(M);a_{3}v^{s}(E);a_{4}v^{s}(S)].$$

### 2.6 Parallel connection Criss-cross attention network

The sequence features extracted by BiLSTM may have a few limits. First, with an increase in sentence length, the feature extraction ability of BiLSTM declines (Huang et al., 2019). In addition, LSTM has been shown to have weaker feature extraction ability than attention mechanism models, such as transformers, when dealing with longer sequence texts (Li et al., 2020b). Second, BiLSTM makes each character contribute equally to the task. In other words, BiLSTM is not good at assigning more weight to some important characters in the text sequence, which is very important for NER. In addition, the strong domain features of kiwifruit-related texts mentioned in Section 2.1.3 also pose challenges to the feature extraction ability of the BiLSTM. In short, the feature extraction ability of BiLSTM must be further improved when solving the problem of kiwifruit-named entity recognition. Therefore, a novel module, parallel connection criss-cross attention network (PCAT), is proposed to mitigate the impact of the above limits with the help of CCNet (Huang et al., 2019). The overall structure of the PCAT is shown in **Figure 3B**.

After the agricultural sentence is processed by BiLSTM, a feature map *X∈R^C×W^* is obtained (*C* represents the dimension of BiLSTM and *W* represents the length of the sentence). In this work, agricultural sentences are regarded as pictures with a channel number of *C* and a size of *W×1*. Therefore, PACT first transforms *X* into a feature map *M∈R^C×W×H^* (the value of *H* is 1) through an unsqueeze operation. Each pixel in the feature map *M* represents a character in the agriculture text.

To obtain richer semantic information. The PCAT uses two different convolutional layers with filter sizes of 1 × 1 and 1 × 3 on *M* to generate two feature maps, *M_1_* and *M_2_*. *M_1_* and *M_2_* are put into the CCNet for processing. To learn more complex features, PCAT applies two convolutional layers with filter sizes of 1 × 3 to *M_1_* and *M_2_*. Finally, *M_1_* and *M_2_* are added, and the output vector of the PCAT *X’∈R^C×W^* is obtained through a squeeze operation.

Using CCNet to calculate the connection between each character, PCAT can assign different weights to different characters to give more attention to key characters. In addition, PCAT can solve the problem of long-distance dependency because it can calculate the degree of association between words in each position and other words that are not affected by distance. Through a parallel structure and convolutional layer, PCAT can obtain richer features from agricultural texts.

### 2.7 Evaluation indicators and experimental environment

#### 2.7.1 Parameter setting

In our proposed model, both the character vector dimension and word vector dimension were set to 50. In the feature encoding layer, the hidden size of both the forward and backward LSTM was set to 300, and to mitigate overfitting, the dropout rate was set to 0.5. For the model training, the batch size was set to 16. Furthermore, the model was trained using stochastic gradient descent with an initial learning rate of 0.0015, and the learning rate decay was set to 0.05. The hyper-parameter configuration of the model is listed in **Table 2**. All experiments were conducted under the conditions listed in **Table 3**.

**Table 2:** Hyper-parameter value.

Parameters	Value	Parameters	Value
character embedding dim	50	learning rate decay	0.05
batchsize	16	LSTM hidden	300
learning rate	0.0015	dropout rate	0.5

**Table 3:** Experimental environment.

Project	Environment	Project	Environment
Operating system	Windows 10(x64)	Hard disk	1T
CPU	i7-10700F@2.90GHz	Python version	3.6.5
GPU	NVIDIA TITANRTX (24GB)	Pytorch version	1.8.1
Memory	64GB	–	–

#### 2.7.2 Dataset division

For dataset division, four datasets were involved in the experiment, namely KIWID, BOSON, ClueNER, and People’s Daily. We obtained the public data according to **Table 2** in study (Liu et al., 2022). This study randomly divided KIWID, BOSON, and ClueNER into training, validation, and test sets according to a ratio of 8:1:1, respectively [refer to Zhang et al. (2021)]. Division of People’s Daily reference https://github.com/zjy-ucas/ChineseNER. The pre-training corpus used in the KIWID-related experiments was the kiwifruit pre-training corpus constructed in this study. The pre-training corpus used in public dataset-related experiments is derived from Lattice-LSTM (Zhang and Yang, 2018), which is pre-trained using Word2vec (Peng et al., 2020) over automatically segmented Chinese Giga-Word. The number of character vectors in the public pre-training corpus is 5.7k, and the number of words in the lexicon is 704.4k.

#### 2.7.3 Evaluation indicators

Precision (*P*), recall (*R*) and F_1_-score (*F_1_*) were used to evaluate the performances of the different models, as shown in Equations (17)-(19).


(17) $$P=\frac{\text{True positives}}{\text{Predictied as positives}}=\frac{T_{P}}{T_{P}+F_{P}},$$

(18) $$R=\frac{\text{True positives}}{\text{Actual positives}}=\frac{T_{P}}{T_{P}+F_{N}},$$

(19) $$F_{1}=\frac{2PR}{P+R}.$$

True positives (*T_P_*) refer to the number of correctly recognized positive samples among all positive samples, whereas false positives (*F_P_*) denote the number of negative samples incorrectly recognized as positive samples. False negatives (*F_N_*) are positive samples incorrectly recognized as negative samples. Among all the positive samples, the more that are predicted correctly, the higher the *P* value. A higher number of positive samples predicted in the testing set yielded a higher *R* value. *F_1_* is the harmonic average of *P* and *R*, providing an evaluation of the comprehensive ability of the model.

## 3 Results

### 3.1 Experiments on KIWID

In this section, some typical NER models such as BiLSTM (Huang et al., 2015), TENER (Yan et al., 2019), LR-CNN (Gui et al., 2019a), LGN (Gui et al., 2019b) and Softlexicon-LSTM (Peng et al., 2020) are considered comparable models. In addition, this section also uses the previous CANER findings JMCA-ADP (Guo et al., 2020) and Att-BiLSTM-CRF (Zhao et al., 2021) as comparison models. Like KIWINER, LR-CNN, LGN and Softlexicon-LSTM are also lexicon-based models. The lexicon used in the experiments in this section are the Kiwifruit lexicon constructed in this study.

The experimental results for KIWID are shown in **Table 4**. It could be observed that the model proposed in this study outperformed other models, and the *F_1_* of this model is at least 0.47 higher than other models, which illustrates the effectiveness of it recognizing kiwifruit-related entities. The performance of our model is significantly improved compared to the baseline model BiLSTM-CRF. This is due to the fact that KIWINER makes full use of kiwifruit lexical information with the help of AttSoftlexicon, and obtains deeper semantic features with the help of PCAT. Compared with CANER models Att-BiLSTM-CRF and JMCA-ADP, KIWNER has achieved obvious improvement, which further verifies the effectiveness of KIWINER. The lexicon-based models LR-CNN, LGN, Softlexicon-LSTM and KIWINER have clear advantages over the rest of the character-based models, illustrating the effectiveness of constructing a kiwifruit-related lexicon and incorporating lexical information into the model.

**Table 4:** Results of each model on KIWID.

Model	P	R	F1
BiLSTM-CRF	84.42	84.54	84.48
Att-BiLSTM-CRF	82.85	88.99	85.81
JMCA-ADP	84.90	90.47	87.59
TENER	86.40	90.19	88.25
LR-CNN	87.08	89.90	88.47
LGN	86.81	89.63	88.19
Softlexicon-LSTM	87.18	89.27	88.21
KIWINER (our)	88.21	90.31	88.94

### 3.2 Experiments on public datasets

To verify the generalization of KIWINER, three public datasets were selected: Boson, ClueNER, and People’s Daily. The experimental results are listed in **Table 5**.

**Table 5:** Results for each model on public datasets.

Model	Boson			ClueNER			People’s Daily		
	P	R	F_1_	P	R	F_1_	P	R	F_1_
LSTM	81.78	72.50	76.86	76.80	71.28	73.94	85.96	82.09	83.98
Att-BiLSTM-CRF	79.93	76.67	78.27	74.73	73.62	74.17	86.28	85.05	85.66
JMCA-ADP	80.10	77.66	78.86	75.82	76.58	76.20	87.96	86.93	87.44
TENER	79.45	81.51	80.47	74.34	77.08	75.68	90.36	90.07	90.22
LR-CNN	84.40	82.04	83.20	80.09	78.47	79.27	91.13	90.74	90.93
LGN	82.16	79.16	80.63	77.01	73.95	75.45	90.75	89.52	90.13
Softlexicon-LSTM	85.75	80.67	83.13	80.50	79.11	79.80	92.31	90.43	91.36
KIWINER	86.96	83.37	85.13	81.05	80.01	80.52	93.23	92.42	92.82

The KIWINER model achieved the best *F_1_* of the three datasets, which were for Boston, ClueNER, and People’s Daily 85.13%, 80.52%, and 92.82%, respectively. The experimental results show that KIWINER not only has performance advantages on the KIWID corpus, but also has a certain generalization in other fields.

### 3.3 Ablation experiments

#### 3.3.1 Effectiveness of new word detection layer

**Figure 5:**
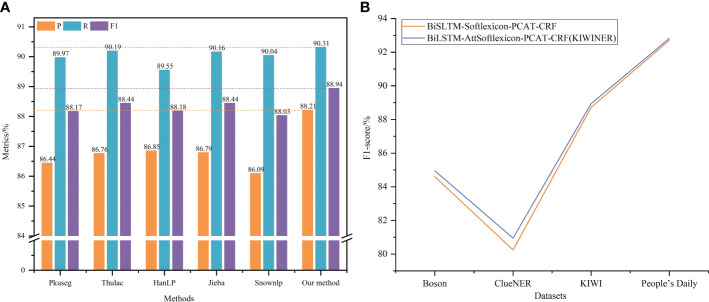
**(A, B)** Results for each lexicon construction method & Effectiveness of AttSoftlexicon.

In the new word detection layer of KIWINER, the adaptability of Jieba to kiwifruit-related texts was enhanced by new word detection and then a lexicon was constructed by word segmentation of kiwifruit-related texts. To verify the effectiveness of this lexicon construction method, this section used several commonly used Chinese automatic word segmentation tools (Pkuseg, Thulac, HanLP, Jieba, and Snownlp) to automatically separate the kiwifruit-related texts collected in this study to construct lexicons and apply them to KIWINER for experiments. Experiments were performed using KIWID. The experimental results are shown in **Figure 5A**.

The method of constructing the lexicon with the aid of new word detection and Jieba achieves the highest *P*, *R*, and *F_1_*, and improves over other methods. This shows that new word detection effectively reduces the negative impact of word segmentation errors on CANER during lexicon construction.

#### 3.3.2 Effectiveness of AttSoftlexicon layer

To verify the effectiveness of the AttSoftlexicon, it was replaced in KIWINER by Softlexicon (Peng et al., 2020), and a comparative experiment was conducted. The experiment used the *F_1_* as the evaluation metric, and the experimental results are shown in **Figure 5B**. The KIWINER model achieved the best *F_1_* for the four datasets. This shows that by assigning different weights to different word set representations, the AttSoftlexicon can help the model to make full use of the position information of characters in its matched words, thus making more full use of lexicon information than Softlexicon.

#### 3.3.3 Effectiveness of PCAT layer

To verify the applicability of the PCAT module for different sequence encoding models, experiments were performed using transformer and GRU instead of BiLSTM. And comparative experiments were carried out with or without the PCAT module in the model. The experiment was divided into three groups, and the results are presented in **Table 6**.

**Table 6:** Application effect of PCAT.

Group	Model	F_1_	Model	F_1_
1	AttSoftlexicon-Transformer-CRF	84.01	AttSoftlexicon-Transformer-PCAT-CRF	85.11
2	AttSoftlexicon-BiGRU-CRF	87.68	AttSoftlexicon-BiGRU-PCAT-CRF	88.85
3	AttSoftlexicon-BiLSTM-CRF	87.17	AttSoftlexicon-BiLSTM-PCAT-CRF(KIWINER)	88.94

The effect of each sequence coding model in the table improved after the introduction of PCAT, indicating the effectiveness and universality of PCAT. The model based on BiLSTM achieved the best effect, which shows the rationality of KIWINER using BiLSTM to encode character sequences.

## 4 Discussion

### 4.1 Comparison of experiments with different variants

**Figure 6:**
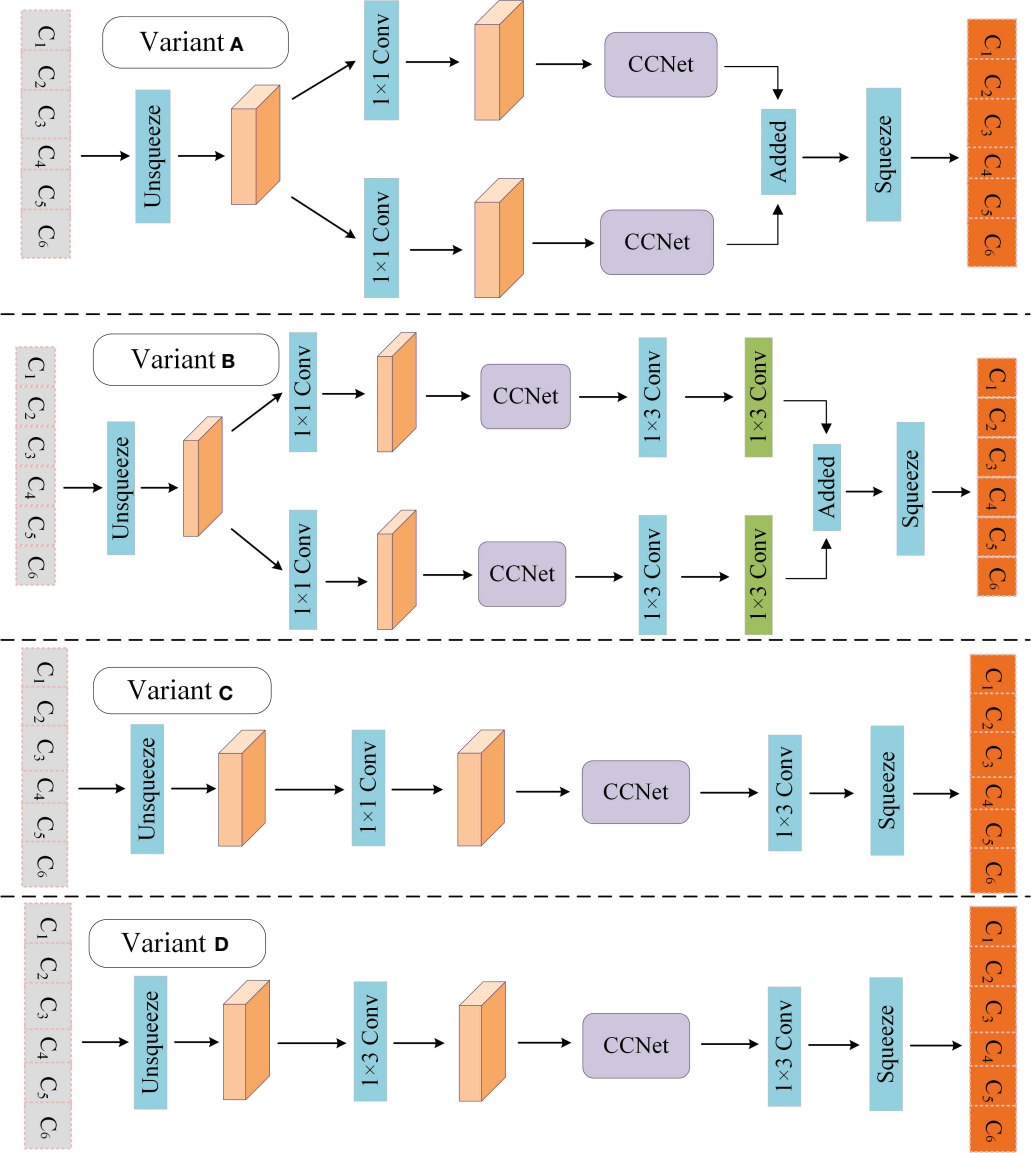
Variants of PCAT.

To verify the rationality of the PCAT module, several variants of it were designed, and the variant was used to replace the PCAT in KIWINER for experiments on Boson, ClueNER, KIWID, and People’s Daily. Variants A and B increased and decreased the depth of the PCAT, respectively. Variants C and D break the parallel connection structure of PCAT. The different variant structures of the PCAT are shown in **Figure 6**. In addition, many researchers use the self-attention mechanism (Self-Att) to improve the feature extraction ability of the sequence encoding layer. In the field of CANER, Guo et al. (2020) introduced a self-attention module after the BiLSTM model to improve the feature extraction ability of sequence coding layer. Therefore, this section refers to the study by Guo et al. (2020) and uses Self-Att instead of PCAT for experiments. Attention unit and head number of Self-Att is 600 and 8. The experimental results are listed in **Table 7**.

**Table 7:** Results for several variants of PCAT.

Module	Boson	ClueNER	KIWID	People’s Daily
Self-Att	83.35	79.73	88.12	91.71
Variant A	84.03	80.26	88.41	92.34
Variant B	84.24	80.41	88.62	92.58
Variant C	84.07	80.32	88.22	92.69
Variant D	84.61	80.34	88.83	92.56
PCAT	**84.95**	**80.95**	**88.94**	**92.82**

The values in bold represent the maximum value in the same column.

Compared with Variants A and B, the PCAT achieved better results, indicating that the depth design of the PCAT is reasonable. Compared with variables C and D, PCAT achieves better results, which shows that a parallel structure can effectively improve the feature extraction ability of the model and help the model obtain richer semantic information. PCAT achieves better results than Self-Att (Guo et al., 2020), which indicates that PCAT is more conducive to improving the model feature extraction capability than the commonly used module Self-Att. PCAT constructs a parallel structure with the help of two different convolutional layers, which allows the model to simultaneously process semantic information from two different perspectives. At the same time, with the help of CCNet, which has good long distance context semantic aggregation capability (Huang et al., 2019), the information can be processed again, and different weights can be given according to different information relationships. Therefore, PCAT can help the model make full use of the feature information input into the model.

### 4.2 Comparative analysis with the previous CANER findings

This section discusses the recognition effects of KIWNER and the previous CANER studys Att-BiLSTM-CRF (Zhao et al., 2021) and JMCA-ADP (Guo et al., 2020) on each category of the kiwifruit dataset KIWID. BiLSTM-CRF (Huang et al., 2015), the baseline model of the above models, also participated in the experiments. The experimental results are shown in **Table 8**, where *F_1_* is chosen as the evaluation metric, and the last column of the table is the running time of each model.

**Table 8:** Entity categories study.

Entity type	BiLSTM	Att-BiLSTM-CRF	JMCA-ADP	KIWINER
KIWI	83.70	80.00	85.90	87.06
DIS	79.17	78.43	81.63	87.50
PEST	77.00	85.71	86.96	89.76
LOC	81.90	82.64	83.69	84.19
PART	94.44	94.23	94.43	96.80
MED	61.64	70.45	73.33	75.28
All category	84.48	85.81	87.59	88.94
Time(s/epoch)	139.14	149.20	144.13	163.87

It can be clearly seen from the table that KIWNER has achieved the best results in each category, especially in disease, pest, pesticide, which contain strong domain features. Although Att-BiLSTM-CRF and JMCA-ADP have made efforts to integrate agricultural features into the model, KIWINER can obtain more agricultural features by using word information with the help of Attsoftlexicon and new word detection. In addition, PCAT can help the model to further make full use of these agricultural features. The category of location related entities usually contain boundary characters, such as “县” (county), “镇” (town), “村” (village), etc., and the category of part related entities have limited diversity and many repeated words, which leads to the recognition difficulty of the above two categories being relatively low. Therefore, KIWINER did not significantly improve the recognition effect of LOC and PART. From the last row of the table, we can see that KIWINER takes more time than other models, which is a disadvantage of KIWINER. KIWNER incorporates lexical information, so it will spend an extra part of time on processing lexical information compared with the character based model. Research on faster character and word matching methods and more efficient sequence encoding modules can be helpful to overcome this shortcoming.

In KIWNER, AttSoftlexicon module and PCAT module both adopt the CCNet model from semantic segmentation, and have achieved good results through experimental verification. With the help of new word detection and AttSoftlexicon, KIWINER incorporate the word information containing domain features into the model. And KIWINER has achieved significant improvement compared with previous which are character-based models. This shows that when solving problems with strong domain features such as CANER, it is a good solution to find a method to integrate more domain features into the model. In addition, the effectiveness of PCAT also shows the importance of making full use of these features.

## 5 Conclusion

To address the lack of an annotation dataset for agricultural named entity recognition in the kiwifruit field, a kiwi-annotated NER corpus KIWID, which contains six categories and 17089 entities was constructed in this study. According to the characteristics of kiwifruit-related texts, a new CANER model, KIWINER, was proposed by statistics-based new word detection and the novel module AttSoftlexicon, PCAT. To alleviate the word segmentation insensitivity caused by the strong specialization of kiwifruit-related texts, statistics-based new word detection was used to enrich the built-in vocabulary of Jieba and improve its applicability to kiwifruit texts to construct the kiwifruit lexicon. Inspired by the CCNet module in the field of semantic segmentation, the AttSoftlexicon was proposed to help the model make efficient use of lexicon information. In addition, this study proposes a PCAT module to improve the feature extraction ability of the sequence coding layer BiLSTM. The experimental results with the comparative models show that our proposed model can effectively improve CANER performance, particularly for difficult-to-recognize categories such as diseases, pests, and farm chemicals.

Moreover, our research can provide reference for developing new deep learning methods for named entity recognition of international texts. Theoretically, our construction method of Attsoftlexicon is also applicable for the named entity recognition of the texts of other similar languages, such as Japanese, Korean etc., which are unnaturally partitioned just like Chinese. In addition, our proposed PCAT module is used to improve the sequence encoding ability of deep learning model essentially. So, applying our proposed PCAT module for the named entity recognition of other language is also theoretically feasible. Therefore, KIWINER can also be used to explore CNER tasks in other crops or other fields with domain features. In the future, we will study how to improve the time efficiency of KIWINER and use it in the construction of kiwifruit Q&A system.

## Data availability statement

The original contributions presented in the study are included in the article/supplementary materials. Further inquiries can be directed to the corresponding author.

## Author contributions

Conceptualization, LZ. Methodology, LZ. Validation, LZ. Formal analysis, LZ and XN. Investigation, LZ, XN, HZ, VG, CR and DN. Data curation, LZ, MZ and MG. Writing—original draft preparation, LZ. Writing—review and editing LZ and DN. Visualization, LZ and XN. Supervision, HZ and DN. All authors contributed to the article and approved the submitted version.

## Funding

This work was supported by the National Key R&D Program of China under grant 2020YFD1100601.

## Acknowledgments

We thank all of the funders and all reviewers.

## Conflict of interest

The authors declare that the research was conducted in the absence of any commercial or financial relationships that could be construed as a potential conflict of interest.

## Publisher’s note

All claims expressed in this article are solely those of the authors and do not necessarily represent those of their affiliated organizations, or those of the publisher, the editors and the reviewers. Any product that may be evaluated in this article, or claim that may be made by its manufacturer, is not guaranteed or endorsed by the publisher.
